# Anal Cancer: The Past, Present and Future

**DOI:** 10.3390/curroncol30030246

**Published:** 2023-03-11

**Authors:** Talha Ashraf Gondal, Noman Chaudhary, Husnaat Bajwa, Aribah Rauf, Duc Le, Shahid Ahmed

**Affiliations:** 1 College of Medicine, University of Saskatchewan, Saskatoon, SK S7N5E5, Canada; tag783@usask.ca (T.A.G.); hub392@usask.ca (H.B.); 2 Faculty of Medicine, St. Klimints Orhidisky School of Medicine, Sofia, Bulgaria, College of Kinesiology, University of Saskatchewan, Saskatoon, SK S7N5B2, Canada; chaudhari@uni-sofia.bg; 3 College of Kinesiology, University of Saskatchewan, Saskatoon, SK S7N5B2, Canada; hza826@usask.ca; 4 Saskatchewan Cancer Agency, Saskatoon Cancer Center, Saskatoon, SK S7N5H5, Canada

**Keywords:** Anal cancer, squamous cell anal cancer, anal canal cancer, human papilloma virus, chemotherapy, radiation, surgery

## Abstract

Anal cancer is a rare cancer that accounts for about 2% of all gastrointestinal tract malignancies. Among anal cancer, squamous cell cancer is the most common malignancy. The incidence of all stages of anal squamous cell cancer has been increasing. Human papillomavirus infection and immunosuppression are major risk factors for anal cancer. The management of anal cancer has evolved over the past several decades and continues to do so. Chemoradiation therapy remains the mainstay for treatment for most patients with early-stage disease, whereas systemic therapy is the primary treatment for patients with metastatic disease. Patients with persistent disease or recurrence following chemoradiation therapy are treated with salvage surgery. Access to novel cytotoxic combinations and immunotherapy has improved the outcomes of patients with advanced disease. This review provides an overview of advances in the management of anal cancer over the past two decades. This paper reviews the epidemiology, risk factors, pathology, diagnosis, and management of localized and advanced anal squamous cell cancer, highlights current knowledge gaps in the management of anal cancer, and discusses future directions.

## 1. Introduction

Anal cancer is among the rare cancers; however, over the last decade, there has been an increase in the incidence by 2.7% annually [1]. Among anal cancer, squamous cell cancer (SCC) is the most common form. Anal cancer has often been the target of much stigma due to its association with sexual behavior and sexual orientation. The management of anal cancer has evolved over the past several decades and continues to do so. From prevention with the human papillomavirus (HPV) vaccine to definitive chemoradiation therapy (CRT) as an organ-preserving approach and, in certain cases, organ-preservation surgery, there is a breadth of options to manage anal canal cancer. Furthermore, with recent advances in systemic therapy, including the use of immunotherapy to manage advanced anal cancer and the use of metastasectomy in selected cases, the treatment landscape for patients with advanced disease is changing. This review provides an overview of the advancements in the epidemiology, risk factors, pathology, diagnosis, staging, treatment, and future directions of anal cancer. The review is focused on anal squamous cell cancer (ASCC).

## 2. Epidemiology

Anal cancer accounts for about 2% of all gastrointestinal tract malignancies [1,2,3]. Throughout the general population of the United States, Western Europe, Australia, and South America, anal cancer is becoming more prevalent, and there has been little change in Asian populations [1,3]. The incidence of anal cancer varies throughout Western nations, with rates in the United Kingdom, The Netherlands, Australia, and the United States ranging from 0.7 to 1.7 per 100,000 people per year. In 2023, 1,958,310 new cancer cases are estimated in the United States, and among them, 9760 (0.5%) are projected to be anal cancer. In the same year, 609,820 cancer deaths are estimated to occur, and 1870 (0.3%) deaths are projected to be from anal cancer [4].

In most nations, women experience higher incidence rates [5]. Based on data from 1973 to 2009 in the Surveillance, Epidemiology, and End Results (SEER) database, there has been a sharp rise in ASCC incidence rates after 1997 [6]. The incidence of ASCC increased by 2.7% per year from 2001 to 2015 [1]. The incidence of all stages of ASCC has been increasing in all demographics—with the fastest annual percentage changes in patients between the ages of 35 and 49 years, followed by those aged 50–64 years [6]. For women, the most prevalent age range is ≥ 65 years, followed by 50–64 years [6]. This trend is believed to be mostly driven by the rise in HPV infection rates.

## 3. Risk Factors

HPV infection is a major risk factor for anal cancer [7]. Sexual behavior and immunosuppression are key drivers of anal cancer risk [8]. HPV is a key risk factor in 80–85% of anal cancer cases; most are associated with HPV16 infections [7]. Because most anal cancer cases are related to HPV infection, factors that increase the risk of persistent HPV infection influence the incidence of anal cancer [7,8]. For example, anal intercourse and a high number of sexual partners correlate with HPV infection. People living with human immunodeficiency virus (HIV) infection, men who have sex with men (MSM), women with HPV-related gynecological cancer or precancerous lesions, and individuals with a solid organ transplant or autoimmune disorders are at high risk of anal cancer [8,9,10,11,12]. In addition, old age, female sex, and smoking have been attributed as risk factors for anal cancer [13,14,15].

Women have a high incidence of anal cancer compared to men. A population-based study examined the trend in ASCC incidence over a 15-year period in the United States and found that about two third of all newly diagnosed cases of ASCC occurred in women [16]. Anal cancer is uncommon in women aged under 40 years, and its incidence in women increases with age [17,18]. Of note, anal cancer is relatively rare compared to the prevalence of HPV infections in women. The Million Women Study, a population-based prospective study, identified the history of cervical intraepithelial neoplasia grade 3 (CIN3), smoking, and previous use of oral contraceptives as risk factors for anal cancer [18].

A meta-analysis calculated comparable estimates of anal cancer incidence in high-risk groups [14]. Overall, the incidence rate of anal cancer among HIV-positive MSM was 85 cases per 100,000 people per year compared with 13 cases per 100,000 people per year for patients with solid organ transplants (Table 1). The use of highly active antiretroviral therapy (HAART) has not decreased the incidence of anal cancer [19]. A systematic review showed that anal cancer incidence has continued to rise in women, especially if they are living with HIV, despite the use of HAART [20]. Because the risk for anal cancer rises with the length of HIV infection, improvements in HIV patient survival have likely contributed to the increased incidence of anal cancer in general.

## 4. Pathology

ASCC represents more than 80% of all anal cancer cases [1,2,3]. Histological confirmation is vital to eliminate several different and uncommon types of anal cancer [21]. Examples of other pathology include adenocarcinoma, lymphoma, gastrointestinal stroma tumors, melanomas, and neuroendocrine tumors [21]. The risk factors and management of anal non-squamous cell cancer are different from ASCC.

### 4.1. Anatomy of Anal Canal

The anus comprises the anal canal and the anal margin. The anal canal extends from the anorectal junction to the anal margin. It is lined by glandular mucosa that transitions to squamous epithelium distally [22]. The anal canal is divided into the proximal two-thirds, and the distal one-third by anal valves called the dentate line. The anal margin is lined with epidermis and is a pigmented skin that extends from the anal opening to a 5 cm radius laterally. Cancer beyond this point is classified as skin cancer, otherwise referred to as perianal cancer [23].

### 4.2. SCC

SCC of the anal canal primarily develops at the transformation zone between squamous and columnar epithelium [2]. The basement membrane surrounding and within the SCC tumor lacks a myoepithelial layer and presents very similar to skin adnexal and salivary gland neoplasms [2]. In over 90% of cases, there is a clear squamous differentiation with keratinization and intracellular bridges [2,24]. ASCC arising above the dentate line is termed nonkeratinizing cancer. Cytokeratin 5 and 6 (CK5 and CK6, respectively) are used to provide evidence of squamous lineage, indicating a diagnosis of SCC and ruling out poorly differentiated adenocarcinoma and well-differentiated neuroendocrine carcinoma [25]. Immunohistochemical staining for p63 protein is highly specific for the diagnosis of SCC [25]. P63 protein resides on the long arm of chromosome 3, which is the site of frequent SCC genomic amplification [25]. It is important to note that variants of SCC, including basaloid (cloacogenic cell), transitional, and spheroidal cancers, are now classified under SCC [24].

#### Gene Expression Profile

Gene expression profiling of ASCC has shown that mutations in phosphoinositol-3-kinase pathway-related genes (PI3K/AKT/mammalian target of rapamycin [mTOR]), MLL2, and MLL3 are relatively common along with differential expression of genes in relation to HPV infection [26,27,28]. Mutations in the tumor suppressor genes TP53 and CDKN2A correlate significantly with HPV-negative cases [26,28]. Programmed death ligand 1 (PD-L1) expression (combined positive score [CPS] ≥ 1) is noted in about 30% of HPV-positive compared with 40% of HPV-negative cases [28]. Furthermore, evidence suggests that patients with HPV-negative and aberrant p53 ASCC have inferior survival compared with patients with HPV-positive and wild-type p53 ASCC [28,29]. However, the predictive role of gene expression profile in tailoring treatment for anal cancer is currently not known.

### 4.3. Adenocarcinoma

Anal adenocarcinoma is uncommon and only accounts for 5–10% of all anal cancer cases [30]. Anal adenocarcinoma is staged like ASCC, but these cases are treated similarly to rectal cancer with concurrent radiotherapy and capecitabine or 5-fluorouracil (5FU) followed by abdominoperineal resection (APR) [30,31].

## 5. Diagnostic Work Up

An early diagnosis of anal cancer is important for a better outcome. A complete history, physical examination, an anorectal exam in both men and women and a gynecological examination in women are important initial assessments. For a more detailed staging, various imaging modalities, including computed tomography (CT) and pelvic magnetic resonance imaging (MRI) with or without positron emissions tomography (PET) or endoanal ultrasound, are important next steps.

### Imaging Modalities

Staging CT and phased-array pelvic MRI are recommended for anal cancer staging [32,33,34,35]. MRI has been reported to be 90–100% sensitive in identifying anal cancer [33,35]. MRI provides specific information regarding the position—whether it is situated in the canal or the margin [33]. MRI shows the circumferential tumor extent and the infiltration of anal cancer in nearby organs and helps detect neoplastic nodes [33,36]. When phased-array MRI is unavailable, endoanal ultrasound can be used to determine the depth of anal cancer in the sphincter complex [32,34].

PET is a very effective and accurate imaging modality used for cancer detection and staging. PET detects tumors based on molecular alterations [37]. Approximately 98% of anal tumors can be detected by PET using 18-fluorodeoxyglucose (FDG) [33]. When used for diagnosis, FDG-PET provides information regarding several important markers for staging: the size of the primary tumor, involved lymph nodes and their status, and the detection of distant metastasis. A systematic review and meta-analysis of 17 studies comparing PET or PET/CT with conventional imaging studies showed that the sensitivity for identifying primary tumors with a PET or PET/CT scan is 99% compared with 67% for CT alone. The overall sensitivity and specificity of PET or PET/CT for detecting inguinal lymph nodes are 93% and 76%, respectively [37]. Of note, PET or PET/CT helped upstage 5.1–37.5% of patients and downstage 8.2–26.7% of patients. Based on the PET results, the treatment plan was modified in 12.5–59.3% of cases [37]. When compared with contrast-enhanced CT, FDG-PET has higher sensitivity for abnormal lymph nodes [33].

## 6. Staging

Tumor node metastasis (TNM) classification is a system of determining malignancy based on the characteristics of the tumor [38]. This system combines three factors—tumor, lymph nodes, and metastasis—for the purpose of indicating the extent of the cancer.

Tumor (T) refers to the size and extent of the primary tumor. A higher number after the T indicates a larger size and growth in nearby tissues [38]. TX indicates the tumor is unable to be measured, and T0 indicates the absence of a tumor. This refers to a high-grade squamous intraepithelial lesion (previously described as carcinoma in situ, anal intraepithelial neoplasia [AIN] II–III, or high-grade AIN). T1 indicates a cancer size of less than 2 cm, T2 as between 2 and 5 cm, T3 as > 5 cm, and T4 as any size but growing into the surrounding organs [38,39].

Lymph node (N) refers to nearby lymph nodes involved with the tumor. NX indicates nearby lymph nodes are unable to be measured, and N0 indicates the absence of cancer in nearby lymph nodes. N1 indicates inguinal, mesorectal, internal iliac, or external iliac nodes. They are further defined as N1a, metastasis in inguinal, mesorectal, or internal iliac lymph nodes; N1b, metastasis in external iliac lymph nodes; and N1c, metastasis in external iliac with any N1a nodes.

Metastasis (M) refers to whether cancer has spread from the original tumor. M1 indicates the presence of distant metastases.

Based on the TNM status, the anal canal is staged I through IV (Table 2). Stages I and II are localized cancer; stage III is locally advanced cancer, and stage IV is metastatic disease. A recent systematic review and meta-analysis of 62 studies involving 10,569 patients with anal cancer over the past three decades suggested that the use of newer imaging modalities in staging may result in misclassification of the nodal stage and a high false positive nodal stage [40].

## 7. Management of Localized and Locally Advanced Disease

APR involves a combined anterior abdominal and perineal approach for the removal of the distal colon, rectum, anus, and anal sphincter complex and forming a permanent colostomy. This approach was the primary treatment for ASCC prior to the 1970s [41]. As a primary treatment for anal cancer, APR is associated with about 3% surgical mortality with 5-year overall survival (OS) of 40–70% [42,43].

In the 1970s, researchers at Wayne State University experimented with preoperative radiotherapy (RT) and chemotherapy with fluoropyrimidine, 5FU, and mitomycin C (MMC) in a small patient cohort that showed a high rate of complete pathologic response, ushering in a new era in the treatment of this disease [44]. In a follow-up study, 45 patients were treated with definitive CRT, with a remarkable complete response rate of 84% [45]. Subsequently, several early single-arm studies using CRT as a definitive treatment for anal cancer, with surgery being reserved for residual or recurrent disease, reported in the early 1990s showed promising results [46,47,48]. The use of definitive CRT as an organ-preservation approach has been confirmed in several phase III randomized clinical trials (RCTs) [49,50,51,52,53,54] Table 3. These trials examined the benefit of definitive CRT versus radiotherapy alone, sequencing and optimal chemotherapy regimens with concurrent radiotherapy, and high-dose radiotherapy.

### 7.1. Radiotherapy versus CRT

The UK Coordination Committee on Cancer Research (UKCCCR) phase III trial involving 585 patients with ASCC compared definitive radiotherapy with CRT [49]. The authors demonstrated that the addition of 5FU and MMC combination chemotherapy to radiotherapy was associated with an overall 46% risk reduction in the local failure rate (RR 0.54, 95% confidence interval [CI] 0.42–0.69), with a local failure rate of 59% with radiotherapy versus 36% with CRT. Furthermore, CRT was associated with a significant reduction in anal cancer mortality, with a three-year anal cancer mortality of 39% with radiotherapy versus 28% with CRT (RR 0.71, 95% CI 0.53–0.95) [49]. The European Organization for Research and Treatment (EORTC) trial randomized 110 patients with anal cancer to radiotherapy or CRT (combination of 5FU and MMC) [50]. CRT was associated with significant improvement in local and regional control rates, with an 18% difference in local and regional failure rates between the groups and a 32% difference in colostomy-free time between the groups.

### 7.2. Chemotherapy Regimen and Sequence

MMC is an important component of the chemotherapy backbone. A randomized phase III trial that compared 5FU combined with MMC versus 5FU alone showed a better colostomy-free rate (71% vs. 59%, P = 0.014) and disease-free survival (DFS) (73% vs. 51%, P = 0.0003) with combination chemotherapy at four years [51]. However, the addition of MMC to 5FU was associated with a greater risk of high-grade toxicity (23% vs. 7% grade 4 and 5 toxicity, P ≤ 0.001). Two key RCTs compared MMC to cisplatin plus 5FU and reported conflicting results [52,53]. The US GI intergroup RTOG 98-11 phases III trial randomized patients with anal cancer to CRT including 5FU and MMC versus two induction cycles of 5FU and cisplatin followed by CRT including 5FU and cisplatin. Compared with the 5FU and cisplatin combination, the 5FU and MMC combination was associated with significantly better 5-year DFS and OS [52]. At five years, DFS was 67.8% for 5FU and MMC compared with 57.8% for 5FU and cisplatin; OS was 78.3% and 70.7%, respectively. However, the ACT II trial employed a 2 × 2 factorial design and evaluated 5FU and cisplatin with 5FU and MMC and two cycles of 5FU and cisplatin for maintenance. Both regimens had similar complete response rates of about 90% with toxicity rates of about 70% and comparable DFS and OS [53]. At three years, progression-free survival (PFS) was 74% with two cycles of maintenance 5FU and cisplatin versus 73% with observation alone (P = 0.70). The 3-year OS rate was 86% for 5FU and MMC and no maintenance, 84% for 5FU and cisplatin and no maintenance, 83% for 5FU and cisplatin and maintenance therapy, and 82% for 5FU and MMC and maintenance therapy.

The ACCORD phase III trial examined the ability of induction chemotherapy with two cycles of 5FU, cisplatin, and radiation dose escalation above 60 Gy to improve colostomy-free survival. The trial failed to demonstrate the benefit of induction chemotherapy or radiation dose escalation [54]. The five-year colostomy-free survival rate, the primary endpoint of the study, was 76.5% with induction chemotherapy versus 75% with no induction treatment (P = 0.37). Similarly, the 5-year colostomy-free survival rate with a high dose after radiation was 77.8% versus 73.7% with a standard dose booster (P = 0.067). The addition of an anti-epidermal growth factor receptor inhibitor, monoclonal antibody cetuximab, to CRT failed to show better outcomes in two phase II trials involving patients with anal cancer with or without HIV infection [55,56].

CRT has been associated with acute adverse effects, including cytopenia, infection, and gastrointestinal and skin toxicities, as well as late adverse effects, including moderate-to-severe fecal incontinence, erectile dysfunction, dyspareunia, infertility, and chronic rectal pain [57,58]. RTOG-0529, a phase II trial that compared intensity-modulated radiation therapy (IMRT) with 5FU and MMC to conventional CRT as per RTOG 9811, showed that IMRT was associated with 26% and 15% improvement in severe dermatologic and gastrointestinal toxicities, respectively, and a 12% improvement in moderate to severe hematological toxicities with comparable long-term outcomes [59,60].

**Figure 1:**
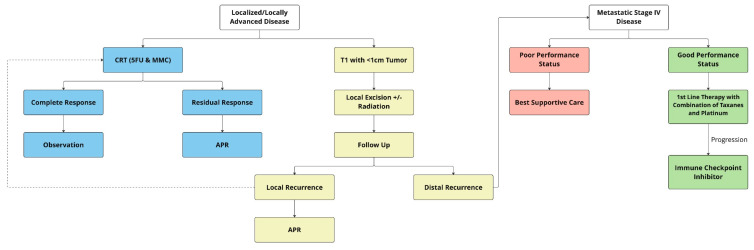
Treatment algorithm of localized and advanced anal cancer. APR = abdominoperineal resection; CRT = chemoradiation therapy.

Taken together, definitive CRT with 5FU and MMC is the best treatment option for localized and locally advanced ASCC, with locoregional control rates of 68–84%, colostomy-free survival rates of 65–75%, and 5-year OS rates of 65–79%. External beam radiation at a dose of 50.4 Gy, with 55–59 Gy for patients with locally advanced disease, including T3/4 tumor or node-positive disease, is recommended with two cycles of 5FU and MMC [52,53] Figure 1. Induction chemotherapy with two cycles of 5FU and cisplatin or maintenance therapy with two cycles of 5FU and cisplatin is not recommended outside the setting of clinical trials. Nonrandomized studies have supported the use of capecitabine as an alternative to 5FU [61,62]. Of note, current trials are evaluating the role of immunotherapy in combination with CRT in reducing the risk of local and distant recurrence.

### 7.3. Surgery

Efforts should be made to treat patients with newly diagnosed anal cancer with CRT as best as possible due to the better outcomes compared with historical data of APR. Surgery with APR and formation of end colostomy has mostly been reserved for the purpose of salvage therapy for patients with disease recurrence, tumor progression following CRT, or for patients who are not a candidate for definitive CRT [21,63,64]. In addition, local excision of low-risk stage I disease is a favorable option. Although perineal colostomy following APR has been studied in patients with low rectal surgery, its use in anal cancer is not well defined [65].

#### Local Excision for Stage I ASCC

Only a small number of individuals with stage I disease have been included in the phase III trials involving CRT. The local excision of T1 tumors has been associated with favorable outcomes and a low risk of complications [66,67,68]. The use of local excision has increased over time in patients diagnosed with T1N0 anal canal cancer, according to a retrospective cohort study that included 2243 people from the National Cancer Database, between 2004 and 2012 (17.3% in 2004 to 30.8% in 2012, P < 0.001) [67]. The 5-year OS rate was 85.3% for patients treated with local excision and 86.8% if they received definitive CRT (P = 0.93). A small observational study reported no recurrence at four years following local excision in patients living with HIV and small well-differentiated ASCC [68]. Nevertheless, a systematic review involving 23 studies, mostly retrospective, examined outcomes of local excision of T1 anal cancer and showed a high 5-year recurrence rate of 37% and OS of 69% [69]. Retrospective data showed that the addition of RT following local excision was associated with a lower locoregional recurrence rate and better survival [70]. However, the level of evidence is low given the absence of prospective, randomized, controlled research.

### 7.4. Special Circumstances

#### 7.4.1. People Living with HIV

Multiple cohort studies have shown that standard CRT with 5FU and MMC is as safe and effective in people living with HIV and using HAART as in patients without HIV [71,72,73,74,75]. Furthermore, newer evidence does not support a negative correlation between low CD4 counts and high CRT-related complications [74]. Hence, patients living with HIV should be treated similarly to patients without HIV. Nevertheless, patients with active HIV-related complications might require dose modification for better treatment tolerance [76].

#### 7.4.2. Patients with Para-Aortic Node Involvement

The optimal management of patients with isolated metastatic para-aortic node involvement is not known. These patients are at high risk of developing additional metastatic disease. Small, single-institution studies have reported that extended-field CRT can potentially be curative with manageable treatment-related toxicity in some patients with para-aortic node involvement [77,78]. Hence, CRT with extended field radiation involving the para-aortic nodes is an appropriate option for patients with good performance status with isolated para-aortic node involvement.

#### 7.4.3. SCC of the Rectum

SCC of the rectum is rare and represents 0.3% of all rectal cancer. Most cases are extensions of ASCC to the rectum or adenosquamous cancer. They are treated similarly to ASCC with definitive CRT using 5FU and MMC [79].

## 8. Management of Recurrent and Metastatic Disease

### 8.1. Salvage Abdominal Perineal Resection for Locoregional Recurrent Disease

About 30% of patients with anal cancer develop locoregional failure that may result in significant morbidity, as well as the risk of distant recurrence and mortality [49,50,51,52,53,54]. Patients with locally advanced disease, including T3/4 tumor or node-positive disease, are at high risk of recurrence. A post hoc analysis of the ACT II trial showed that poor compliance to CRT, including dose reduction or omission of cycle two of chemotherapy and treatment delay for > 42 days, were associated with high recurrence [80]. In addition, residual HPV circulating tumor DNA (ctDNA) following CRT is a risk factor for recurrence [81].

Locoregional relapse may be treated with salvage abdominal perineal resection, which results in attaining local pelvic control in about 60% of all cases. Salvage surgery also has a 5-year OS rate of 30–60% [21,63]. A systematic review and pooled analysis of 39 observational studies, including 1388 patients, reported a 5-year DFS rate of 38.3% and OS rate of 45% following salvage surgery for recurrent disease with major complications rates and postoperative mortality rates of 27.7% and 1.7%, respectively [64]. Radiotherapy has a limited role in patients with locoregional recurrence following CRT. Likewise, the benefit of pre- or postoperative systemic therapy, including the use of chemotherapy or immunotherapy in reducing the risk of recurrent disease, is not known and warrants further research.

### 8.2. Systemic Therapy for Metastatic Anal Canal Cancer

#### 8.2.1. Chemotherapy

Systemic therapy is the mainstay of treatment for patients with de novo metastatic cancer or those who develop recurrent metastatic disease. Overall, 10–20% of patients tend to experience distant relapse after CRT, and < 10% of patients present with de novo metastatic disease [21]. For several decades, in the absence of clinical trials combination of cisplatin plus 5FU remained the preferred first-line therapy for metastatic ASCC [82]. Although a higher response rate was noted with a combination of 5FU and cisplatin in chemotherapy-naïve patients, most were of short duration [83,84]. An international multicenter randomized phase II trial compared carboplatin plus paclitaxel with cisplatin plus 5FU in 91 patients with ASCC and showed that carboplatin plus paclitaxel had significantly better PFS (5.7 vs. 8.1 months) and OS (12.3 vs. 20 months) [85]. Furthermore, cisplatin and 5FU produced significantly more treatment-related complications compared with carboplatin and paclitaxel. Based on the results of this trial, a combination of carboplatin plus paclitaxel is considered to be one of the first-line standard chemotherapy regimens for patients with advanced ASCC (Figure 1).

A French phase II trial evaluated the efficacy and safety of docetaxel, cisplatin, and 5FU (DCF) in 69 treatment-naïve patients with advanced ASCC [86]. A modified regimen (mDCF) was recommended in older patients. Both standard and mDCF demonstrated similar benefits, including response rates of 83% versus 89% and a median PFS of 11 months in both groups. However, patients treated with mDCF experienced a durable response and better treatment tolerance. Clearance of HPV ctDNA level following DCF is an important prognostic factor [87].

Taken together, combination therapy with the use of platinum-containing compounds and taxanes—either combination of carboplatin plus paclitaxel or mDCF—is an effective regimen in patients with newly diagnosed advanced ASCC. This approach produces a high objective response rate, PFS of > 6 months, and is the preferred option in patients with good performance status.

#### 8.2.2. Immunotherapy

Immune checkpoint surface receptors, such as programmed cell death protein 1 (PD-1) and cytotoxic T-lymphocyte-associated protein 4 (CTLA-4), are expressed by tumor cells to avoid antitumor activity by T cells [88]. Several PD-1 or PD-L1 inhibitors have produced persistent clinical responses in a variety of solid tumors, including malignancies associated with viruses, such as HPV, but also malignancies not associated with viruses [88]. Immune checkpoint inhibitors have shown efficacy in patients with unresectable metastatic SCCA [89,90,91]. A phase II clinical trial evaluated the role of the anti-PD-1 monoclonal antibody nivolumab in 37 patients with surgically unresectable or metastatic ASCC regardless of PD-L1 expression; they had received at least one prior line of systemic therapy. The objective response rate was 24%, with no serious adverse events reported [90]. The median PFS was 4.1 months, and the 6-month PFS rate was 38%. The median OS was 11.5 months, with a 1-year OS rate of 48% [90]. In the KEYNOTE-028 basket trial, a multicenter phase Ib study with 20 cohorts of patients with PD-L1-positive advanced solid tumors, the efficacy of pembrolizumab, another anti-PD-1 monoclonal antibody, was evaluated in 24 patients with advanced ASCC who had a PD-L1-positive tumor and had failed one prior standard therapy. The study revealed an objective response rate of 17% in the overall group [91]. The disease control rate was 58%.

In KEYNOTE-158, a global phase II trial, 112 patients with advanced ASCC who had failed or were intolerant to standard therapy—67% had PD-L1-positive tumors—were treated with pembrolizumab. The response was 15% in patients with PD-L1-positive tumors and 3% in patients with PD-L1-negative tumors. Overall, 18% of patients experienced grade 3–4 adverse events [89]. There were no treatment-related deaths. The median PFS was 2.1 months, but the median OS was 11.9 months. The SCARCE trial evaluated the role of a checkpoint inhibitor in combination with chemotherapy as a first-line therapy in patients with anal cancer [92]. In this randomized phase II trial, 97 patients with treatment-naïve metastatic or locally advanced ASCC were randomized 1:2 to mDCF or mDCF plus atezolizumab, a PD-1 inhibitor. There were no differences in the outcomes of the two arms. The response rates were 74.6% versus 78.1%, the 12-month PFS rate was 44.2% versus 43.2%, and the 12-month OS rate was 77.7% versus 80.8% with mDCF versus mDCF plus atezolizumab. Early-phase trials have examined the role of adoptive T-cell therapies and modified T-cells and have shown durable responses and required further investigation [93].

Overall, immune checkpoint inhibitors benefit a subset of patients with unresectable or metastatic ASCC who progress on prior chemotherapy. However, its role in combination with chemotherapy of treatment-naïve patients is unknown. Checkpoint inhibitors are an appropriate option for patients with previously treated advanced ASCC. Nevertheless, additional controlled trials are required to establish immunotherapy as the mainstay of treatment for anal cancer.

#### 8.2.3. Targeted Therapy

Various targeted therapies have been evaluated in patients with advanced anal cancer [93]. In CARACAS, a phase II study, 60 patients with treatment-refractory ASCC were randomized to the PD-L1 inhibitor avelumab or the anti-epidermal growth factor receptor (EGFR) monoclonal antibody cetuximab plus avelumab and showed a response rate of 17% versus 10%, a disease control rate of 57% versus 50%, and median PFS of 3.9 versus 2.0 months, favoring the combination [94]. However, the median OS was 7.8 months with the combination versus 13.9 months with the anti-PD-L1 inhibitor.

A phase II study that evaluated the combination of atezolizumab, a PD-L1 inhibitor, and bevacizumab, an anti-vascular endothelial growth factor, in previously treated patients showed a response rate of 11%, median PFS of 4.1 months, and OS of 11.6 months [95]. However, 35% of patients developed grade 3/4 adverse events. The results with anti-EGFR monoclonal antibodies, anti-VEGF monoclonal antibodies, and other targeted therapies are preliminary and require further investigation.

### 8.3. Metastasectomy

The para-aortic nodes, liver, lungs, and skin are among the most common metastatic sites in anal cancer [80]. Systemic therapy is the standard treatment for these patients. Currently, there are scarce data, mostly retrospective, regarding the benefit of metastasectomy in stage IV anal cancer [83,96,97]. A review of the National Cancer Database of > 2000 patients with metastatic anal cancer from 2004 to 2014 revealed better outcomes for patients with liver metastasis who underwent metastasectomy with a median OS of 34 versus 16 months (P < 0.0001) [97]. There was no difference in survival for metastasectomy of other sites. Other retrospective studies have also reported better outcomes for patients with metastatic ASCC if they had received multimodal treatment, including metastasectomy and/or ablation [83,96]. Metastasectomy or other liver-directed therapy may be considered in selected patients with good performance status and limited liver metastases.

## 9. Surveillance and Survivorship

After completion of CRT, the initial evaluation is recommended at 3 and 6 months after starting treatment to assess complete clinical response. A post hoc analysis of the ACT II trial showed that 72% of patients who had not achieved a complete clinical response at 11 weeks from the start of CRT experienced a complete clinical response by 26 weeks from the start of CRT [98]. Due to delayed remission, if the disease continues to persist, a biopsy can be deferred up to 6 months after CRT [63]. Patients who fail to achieve a complete response within six months of completing CRT require salvage surgery. Once a complete clinical response is attained, monitoring every 3–6 months for two years and then every 6 months for three years is recommended [99,100].

Assessment includes digital rectal examination (DRE) and palpation of inguinal lymph nodes. The assessment can be complemented with an anoscopy or proctoscopy during the first three years. An annual CT scan of the chest, abdomen and pelvic region may be considered yearly over a span of three years in patients with locally advanced disease with T3-T4 or node-positive disease. The results from the ACT II trial indicate a relapse of < 1% after three years [99]. Suspicious lesions require biopsy and further imagining techniques, such as MRI or PET. Compared with imaging techniques, clinical methods allow for a more in-depth measurement of disease recurrence or persistence, as well as regional relapse [63]. The periodic assessments are important for the surveillance of disease recurrence and provide an opportunity to address other components of survivorship care, including management of chronic treatment-related complications, sexual health, and psychosocial well-being [101,102].

## 10. Prevention and Screening

HPV are non-enveloped DNA viruses and the single most important risk factor for ASCC [103,104]. AIN is a precursor dysplastic lesion that has been noted to be associated with HPV16, HPV18, and HPV31 [103]. AIN is very prevalent in men living with HIV who present CD4+ cell counts below 500 cells/mm^3^ [103]. Evidence shows that the HPV vaccine has higher efficacy and is cost-effective in a younger population. AIN of grade 2 or 3 is not yet routinely screened for or treated to lower anal cancer risk. The greatest long-term strategy for lowering the risks of both anal cancer and anal condyloma is HPV vaccination [105].

An RCT involving 602 healthy MSM examined the safety and effectiveness of a quadrivalent HPV vaccine against AIN caused by HPV6, HPV11, HPV16, or HPV18 infections. The study showed vaccine efficacy of about 75% in preventing HPV-related grade 2 or 3 AIN in participants who were treated as per protocol with no safety concerns [106]. Another international RCT demonstrated the efficacy and safety of early and catch-up quadrivalent HPV vaccination in 1803 MSM and heterosexual men in reducing HPV6- and HPV11-related external genital warts and HPV6-, HPV11-, HPV16-, or HPV18-related external genital lesions and anal dysplasia over a 10-year period [107]. A Cochrane meta-analysis of 20 RCTs involving 31,940 participants showed comparable immunogenicity of two- and three-dose HPV vaccination, and nonavalent and quadrivalent vaccines offer similar efficacy [108].

The Centers for Disease Control and Prevention’s Advisory Committee on Immunization Practices recommends HPV vaccination between the ages of 11 and 12 years [105]. Females through the age of 26 years, boys through the age of 21 years, and certain specific populations through the age of 26 years are recommended for catch-up immunization.

High-grade squamous intraepithelial lesions (HSILs) are precursor lesions for ASCC. An RCT involving 4459 patients with HIV showed that treatment of HSILs was associated with a 57% reduction in the rate of progression to ASCC compared with active monitoring [109]. Screening for anal cancer and other HPV-related anal and perianal dysplasia that is modeled after cervical cancer screening is recommended by some jurisdictions for patients living with HIV who are ≥ 35 years, regardless of HPV vaccination status [110]. Currently, the cost-effectiveness of screening of healthy women for anal cancer is lacking. It may be considered in women who are at high risk for anal cancer, including those who are immunosuppressed or with a history of a genital tract malignancy or those who are infected with HPV demonstrated on Pap smears [17].

## 11. Future Directions

Despite treatment advances for anal cancer, there are several knowledge gaps in the management of both early and advanced anal cancer. Research directed at prevention is important to reduce the rising burden of ASCC. For patients with early-stage disease, there is a need for biomarker-driven clinical trials to identify patients who would benefit from treatment de-escalation or those who would need more intense treatment to reduce the risk of recurrence. At the same time, research focusing on minimizing the late effect of CRT in patients with an early stage of the disease is key to improving quality of life and survivorship care. Trials are evaluating the role of maintenance immunotherapy in high-risk patients following CRT, as well as radiation dose escalation or de-escalation. For patients with advanced disease, there is an unmet need for novel agents using genomic analysis to improve outcomes. Despite early negative results, the identification of patients who would benefit from combination immunotherapy and chemotherapy is a key step in managing advanced anal cancer. Currently, there are multiple ongoing clinical trials concerning the management of anal canal cancer that will address various questions (Table 4).

## 12. Conclusions

Although anal cancer is rare, its incidence is rising. ASCC is the most prevalent form of anal cancer, and most cases are related to HPV infection. Overall, the management of anal cancer has seen advances over the last two decades. However, CRT with 5FU and MMC has remained the current standard therapy for early and locally advanced disease. The role of targeted or immunotherapy in combination with CRT or alone is currently not known. A small subset of patients with low-risk T1 cancer may be treated with local excision. About 20–30% of patients develop recurrent disease and require a salvage abdominal perineal resection. Combination chemotherapy with a platinum-containing compound and taxanes is the mainstay of treatment for patients with metastatic disease. Upon progression, a checkpoint inhibitor is a standard option. Liver metastasectomy may be considered in selected patients with limited liver metastases. HPV vaccine is an important long-term strategy for lowering the risks of HPV-related anal cancer. Due to treatment-related morbidity and recurrence, it is important to individualize treatment, including treatment escalation or de-escalation, to improve the outcomes of patients with anal cancer.

## Figures and Tables

**Table 1:** Incidence rates of squamous cell anal cancer in high-risk groups.

Variables	Incidence Rates (95% Confidence Interval [CI]): Cases Per 100,000 Person-Years
HIV-positive MSM	85 (82–89)
non-MSM male PLHIV	32 (30–35)
Female PLHIV	22 (19–24)
HIV-negative MSM	19 (10–36)
Women with HPV-related gynecological precancerous lesions or cancer	
Vulvar	48 (38–61)
Cervical	9 (8–12)
Vaginal	10 (3–30)
Solid organ transplant recipients	13 (12–15)
Patients with autoimmune diseases	
Systemic lupus erythematosus	10 (5–19)
Ulcerative colitis	6 (3–11)
Crohn’s disease	3 (2–4)

MSM = men who have sex with men; PLHIV = persons living with HIV; HPV = human papillomavirus.

**Table 2:** Stage of anal cancer as per tumor nodal and metastasis status.

Stage	Tumor	Node	Metastases
0	Tis	0	0
I	T1	0	0
IIA	T2	0	0
IIB	T3	0	0
IIIA	T1–2	1	0
IIIB	T4	0	0
IIIC	T3–4	1	0
IV	Any T	Any N	M1

**Table 3:** Key phase 3 trials evaluated radiation versus chemoradiation therapy, induction or maintenance chemotherapy, or high or standard dose booster radiation therapy in patients with stage 1–3 and all canal cancer.

Trial	N	Arms	Accrual Period	Follow Up Months	Response	Local Failure	Survival
UKCCR [49]	585	RT vs. CRT	1987–1991	42	Good clinical response at 6 wks: 91% vs. 92% (CR 30% to 39%)	59% vs. 36% (P < 0.0001)	3-year anal cancer mortality (39% vs 28%) p = 0.02 3-year OS 58% vs 65% p = 0.25
EORTC [50]	110	RT vs. CRT	1987–1994	60	CR 54% vs. 80%	18% difference in loco-regional failure (0.002)	5-year survival 56% for whole group
RTOG 87-04/EORTC 1289 [51]	310	MMC plus 5FU or 5FU alone	1998–1991	48	Post-treatment positive biopsies 15% vs. 7.7% (p = 0.13)	Colostomy rates (9% vs. 22%; P = 0.002)	DFS favoring MMC 73% vs. 51%; P = 0.0003
RTOG 98–11 [52]	682	5FU-MMC vs. induction 5FU-CDDP for 2 cycles followed by concurrent 5FU-CDDP	1998–2005	60	NR	20% vs. 26.4% (p = 0.08)	5-yr CFS, 71.9% vs. 65.0% (p = 0.05) 5-year DFS, (67.8% vs. 57.8%; P = 0.006) 5-year OS, 78.3% vs. 70.7%; P = 0.026).
ACTII [53]	940	2 × 2 MMC vs. 5FU-CDDP Maintenance 5FU-CDDP for 2 cycles vs. no-maintenance	2001–2008	61	90.5% with MCC vs. 89.6% with cisplatin	Colostomy rates 11–16 (p = NS)	3-year PFS 70% vs. 69% p = 0·70 with maintenance 3-year PFS 69% vs. 69% p = 0·NS with CPDD vs. MMC
ACCORD [54]	307	2 × 2 Induction chemo vs. no induction High dose boost vs. standard doses boost	199–2005	50	CR or major response with sphincter preservation 92% ICT/SD, 97% ICT/HD, 86% CRT/SD, 94% for CRT /HD		5-year DFS70% ICT/SD; 78% ICT/HD; 67% CRT/SD; 68% CRT /HD 5-year OS 74.5% ICT 71% no ICT

CRT = chemoradiation therapy; CDDP = cisplatin; CFS: Colostomy free survival; DFS = disease-free survival; Good clinical response = complete or >50% reduction in tumor; HD = high dose; ICT = induction chemotherapy; MMC= mitomycin; NR = not reported; NS= not reported; OS = overall survival; RT = radiation therapy; SD = Standard dose.

**Table 4:** Current Clinical Trials in prevention and treatment of anal cancer.

NCT Number	Title	Interventions	Study Type	Phase
NCT02369939	Effects of Deep Regional Hyperthermia in Patients With Anal Carcinoma Treated by Standard Radiochemotherapy	•Radiation: Irradiation •Drug: Mitomycin C •Drug: 5–Fluorouracil •Procedure: Hyperthermia	Interventional	Phase 3
NCT03233711	Nivolumab After Combined Modality Therapy in Treating Patients With High Risk Stage II-IIIB Anal Cancer	•Biological: Nivolumab •Other: Patient Observation	Interventional	Phase 3
NCT04444921	EA2176: Phase 3 Clinical Trial of Carboplatin and Paclitaxel +/−Nivolumab in Metastatic Anal Cancer Patients	•Drug: Carboplatin •Biological: Nivolumab •Drug: Paclitaxel	Interventional	Phase 3
NCT02526953	Efficacy Study of Chemoradiotherapy With or Without Paclitaxel in Squamous-cell Anal Carcinoma Patients	•Drug: Paclitaxel •Drug: Capecitabine •Drug: Mitomycins •Radiation: Radiotherapy	Interventional	Phase 3
NCT05374252	Chemoradiotherapy Combined With or Without PD-1 Blockade in Anal Canal Squamous Carcinoma Patients	•Drug: PD-1 inhibitor •Radiation: concurrent chemoradiotherapy	Interventional	Phase 3
NCT04472429	Carboplatin-paclitaxel With Retifanlimab or Placebo in Participants With Locally Advanced or Metastatic Squamous Cell Anal Carcinoma (POD1UM-303/InterAACT 2).	•Drug: carboplatin •Drug: paclitaxel •Drug: retifanlimab	Interventional	Phase 3
NCT04635423	Efficacy, Immunogenicity, and Safety Study of the 9vHPV Vaccine in Japanese Males (V503-064)	•Biological: V503 •Other: Placebo	Interventional	Phase 3
NCT04269369	Implementation of Pre-emptive Geno- and Phenotyping in 5-Fluorouracil- or Capecitabine-treated Patients	•Drug: 5-Fluorouracil	Interventional	Phase 4
NCT04534075	Dietary Fiber During Radiotherapy—a Placebo-controlled Randomized Trial	•Biological: Capsules containing either dietary fiber or placebo	Interventional	Phase 3
NCT04055142	Clinical Trial for Evaluating the Efficacy and Safety of Electrocoagulation vs Topic Sinecatechins vs Topic Cidofovir Within the Treatment to High-grade Anal Intraepithelial Neoplasia in HIV Homosexual Males	•Procedure: electrocoagulation •Drug: cidofovir 1% topical ointment •Drug: sinecatechins 10% topical ointment	Interventional	Phase 3
NCT05662020	A Study to Evaluate the Immunogenicity and Safety of HPV Vaccine in Healthy Female Participants Aged 9–26 Years in China	Biological: Recombinant Nonavalent (types 6/11/16/18/31/33/45/52/58) Human Papillomavirus (HPV) Vaccine (Escherichia coli) Biological: GARDASIL ^®^ 9	Interventional	Phase 3

## Data Availability

Not applicable.
